# Phenotyping patients with a low-flow, low-gradient aortic stenosis and a preserved ejection fraction undergoing transcatheter aortic valve implantation

**DOI:** 10.1093/eschf/xvag146

**Published:** 2026-05-31

**Authors:** Kees H van Bergeijk, Ferdinand Roski, Constantijn S Venema, Tobias Rheude, Bob Ophuis, Jasper Tromp, Yoran M Hummel, Wouter Ouwerkerk, Ad F M van den Heuvel, Hendrik W van der Werf, Yvonne L Douglas, Marianna Adamo, Adriaan A Voors, Teresa Trenkwalder, Michael Joner, Joanna J Wykrzykowska

**Affiliations:** Department of Cardiology, University of Groningen, University Medical Centre Groningen, Groningen, The Netherlands; Department of Cardiovascular Diseases, TUM University Hospital German Heart Center, Munich, Germany; Department of Cardiology, University of Groningen, University Medical Centre Groningen, Groningen, The Netherlands; Department of Cardiovascular Diseases, TUM University Hospital German Heart Center, Munich, Germany; Department of Cardiology, University of Groningen, University Medical Centre Groningen, Groningen, The Netherlands; Department of Cardiology, University of Groningen, University Medical Centre Groningen, Groningen, The Netherlands; Saw Swee Hock School of Public Health, National University of Singapore, and the National University Health System, Singapore, Singapore; Us2.ai, Singapore, Singapore; Department of Dermatology, University of Amsterdam Medical Centre, Amsterdam, Netherlands; Department of Cardiology, University of Groningen, University Medical Centre Groningen, Groningen, The Netherlands; Department of Cardiology, University of Groningen, University Medical Centre Groningen, Groningen, The Netherlands; Department of Cardiothoracic Surgery, University of Groningen, University Medical Centre Groningen, Groningen, the Netherlands; Department of Medical and Surgical Specialties, Radiological Sciences, and Public Health, University of Brescia, Brescia, Italy; Department of Cardiology, University of Groningen, University Medical Centre Groningen, Groningen, The Netherlands; Department of Cardiovascular Diseases, TUM University Hospital German Heart Center, Munich, Germany; Department of Cardiovascular Diseases, TUM University Hospital German Heart Center, Munich, Germany; Department of Cardiology, University of Groningen, University Medical Centre Groningen, Groningen, The Netherlands

**Keywords:** TAVI, HFpEF, Low-flow low-gradient, Symptoms, Cluster analysis

## Abstract

**Background:**

In patients undergoing transcatheter aortic valve implantation (TAVI), low flow, low-gradient (LF-LG) often reflects reduced left ventricular ejection fraction (LVEF). However, characteristics and causes of worse outcomes in patients with paradoxical LF-LG and preserved ejection fraction (pEF) remains poorly understood.

**Objectives:**

To phenotype LF-LG pEF patients using unsupervised echocardiographic clustering.

**Methods:**

We included 827 patients undergoing TAVI (UMCG, the Netherlands). LF-LG pEF was defined as LVEF ≥50%, aortic valve area: ≤1.0 cm^2^, mean gradient <40 mmHg, and a stroke volume index <35 ml/m^2^. Principal component analysis on 323 AI-assessed echocardiographic parameters (US2.ai) was followed by K-means clustering, with internal (*n* = 405) and external (*n* = 173) (TUMunich, Germany) validation.

**Results:**

216/628 patients with complete data (34%) had LF-LG pEF (mean age of 78.8 (± 7.19) years, 57.9% female). Two clusters were identified: cluster 1 (*n* = 77) showed distinct echocardiographic features related to HFpEF, including larger left and right atrial dimensions (volume, area, length, width, and circumference), and impaired left ventricular global longitudinal strain and a reduced left atrial reservoir strain (across multiple views). They were older and had higher rates of atrial fibrillation (AF) and heart failure (HF), and higher HFpEF scores (49.4% H2FPEF score ≥6 and 71.4% HFA-pEFF score ≥5), versus cluster 2 (*n* = 139). Cluster 1 had higher 1- and 5-year cardiovascular mortality and HF-hospitalization risk, comparable to that of classical LF-LG patients. Validation reproduced theseresults.

**Conclusion:**

One-third of TAVI patients with LF-LG aortic stenosis have an typical HFpEF/AF phenotype and a poor prognosis. They may benefit from guideline-directed HFpEF therapy, including SGLT-2 inhibitors and MRA.

## Graphical Abstract

**Graphical Abstract:**
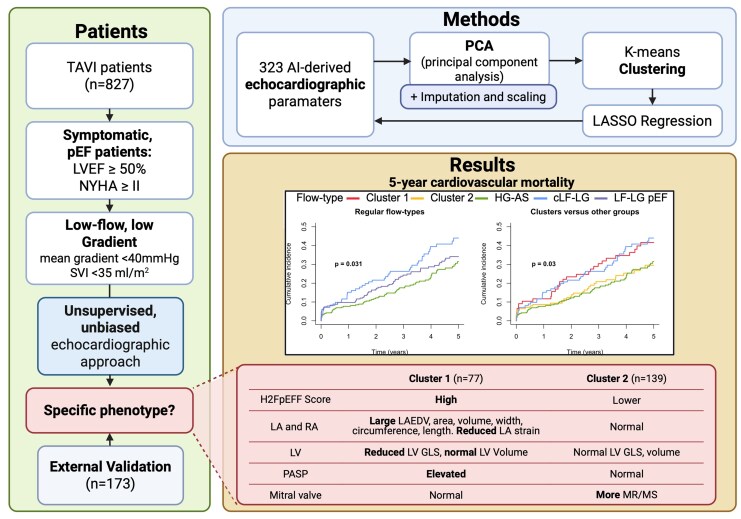
Figure showing the main results of this manuscript, aiming to phenotype patients with a low-flow, low-gradient aortic stenosis with a preserved ejection fraction undergoing TAVI. It contains a short overview of the included patients, methods, and main results.

 

### Condensed abstract

While it is known that a proportion of patients undergoing TAVI have a paradoxical LF-LG pEF aortic stenosis, it is less well understood why this leads to worse outcomes. Therefore, we aimed to phenotype patients with this LF-LG pEF based on unsupervised echocardiographic clustering. 216 (26.1%) patients had an LF-LG pEF status. Two clusters were identified (cluster 1, *n* = 77 and cluster 2, *n* = 139). While cluster 1 included patients with typical HFpEF characteristics, cluster 2 patients were more similar to high gradient aortic stenosis. Patients in cluster 1 had worse outcomes at 1- and 5-year follow-up and could potentially benefit from guideline-directed medical therapies for patients with HFpEF, including SGLT-2 inhibitors and the MRA. These clustering results were validated, both internally and externally.

## Introduction

Severe aortic stenosis (AS) is typically characterized by an elevated transvalvular mean gradient (MG) and reduced aortic valve area (AVA).^1^ In recent years, however, it has become clear that a subset of patients presents with discordant measurements: a small AVA but a low MG AS and low stroke volume index (SVI) <35 mL/m^2^, a condition commonly referred as a low-flow, low-gradient (LF-LG) AS.^2–4^ It is a common assumption that this LF-LG status reflects a late stage AS, with greater extends of cardiac damage and left ventricular damage with a reduced ejection fraction. Paradoxically, a clinically relevant subgroup of patients with a LF-LG AS has a preserved ejection fraction (LF-LG pEF). The phenotype of patients with this paradoxical LF-LG aortic stenosis is less well described. It is commonly stipulated that this group has similar pathophysiological mechanisms to heart failure with a preserved ejection fraction (HFpEF), other concomitant valvular diseases, or atrial fibrillation (AF).^5–8^ The exact phenotype of these patients is thus heterogeneous and not fully understood.

The preferred procedure to treat AS in elderly and high-risk patients has become transcatheter aortic valve implantation (TAVI).^1,9^ While outcomes after TAVI are excellent and have improved over recent years, there are still patients who do not improve symptomatically or have adverse events, which shows an association with the presence of a low gradient AS.^3^ Adequate phenotyping of these LF-LG pEF patients might aid in better patient selection and guide optimal (medical) therapy pre- and post-TAVI. The latter can contribute to a better-informed decision-making, thereby helping to manage expectations of both patients and clinicians, regarding symptom improvement and prognostic benefits.

We hypothesize that distinct echocardiographic phenotypes within LF-LG pEF exist and that one or more phenotypes would be associated with worse clinical outcomes after TAVI. Therefore, we aimed to phenotype these LF-LG pEF patients undergoing TAVI by an unsupervised clustering approach, based on (AI-assessed) echocardiographic images, and explore the impact of baseline characteristics, biomarkers, and HFpEF on both 1-year and 5-year outcomes in a tertiary TAVI centre based in the Netherlands. To assess reproducibility and generalizability, we validated the clustering results internally in a temporary distinct cohort and externally in an independent cohort from a tertiary centre in Germany.

## Methods

### Study design

We included all TAVI-treated patients in a tertiary centre in the Netherlands (University Medical Centre Groningen (UMCG), Groningen). The derivation cohort included patients treated between 2009 and 2020, while the results were internally validated in a temporally distinct cohort of patients from the same centre (2020–2024). To evaluate reproducibility and generalizability, an external validation was performed in a subset of patients treated in the Technical University of Munich (2020–2024). The same inclusion and exclusion criteria were applied to all cohorts. Exclusion criteria were missing aortic stenosis-related echocardiographic parameters (aortic valve (AV) MG, SVI, and left ventricular ejection fraction (LVEF)), previous aortic valve replacement, and an indication other than severe native AS. Approval for this study was obtained from the local research ethical committee (local number: 202100641) following the 1964 Declaration of Helsinki guidelines.

**Figure 1:**
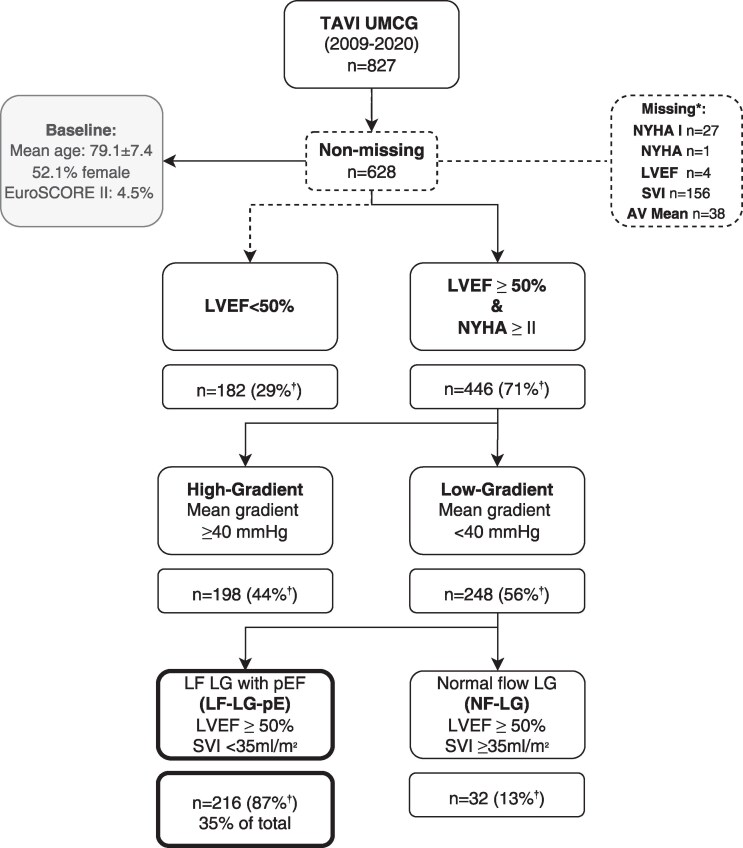
Flow diagram inclusion. Flow diagram shows included patients after excluding patients with missing parameters and subdividing by left ventricular ejection fraction, New York Heart Association, gradient and stroke volume index, resulting in a subset of *n* = 216. † Showing percentage of group from the previous step. *Individual patient can have more than 1 missing variable, resulting 199 patients with one ore more missing variables.

The primary analysis included patients with an LF-LG pEF phenotype, defined by AVA: ≤1.0 cm^2^ (or indexed AVA <0.6 cm^2^/m^2^), LVEF ≥50%, and low AV MG <40 mmHg and a SVI <35 ml/m^2^ (*Figure 1*). In addition, patients had to be in New York Heart Association (NYHA) Class ≥II. The definition of other aortic stenosis types can be found in the supplementary methods.

### Data collection and management

At baseline, characteristics were prospectively collected at the treating centres and aggregated retrospectively. The baseline visit was performed (within) 1 month before the initial TAVI procedure and performed by a medical professional. Follow-up visits were conducted according to the local protocol at the treating centre after 30 days and at the referring hospitals 6 and 12 months after the TAVI procedure. Follow-up data were retrospectively collected from electronic patient records at both treating and referring hospitals. While both centres have different follow-up standards and local protocols, data were retrospectively harmonized.

Transthoracic echocardiography was performed before TAVI according to local guidelines. The echocardiographic images in the derivation cohort were analyzed with a fully automated, validated and clinically available deep learning-based echocardiography software (Us2.ai, version 2.0.0).^10^ Raw echocardiographic DICOM images are analyzed with a convolutional neural network that accurately identifies 2D or Doppler views and segments and annotates cardiac structures. The software provides all standard echocardiographic parameters. Prior to utilizing the derived parameters, multiple confidence checks are conducted to verify the software’s accuracy and reliability. In the external validation cohort, echocardiographic data were obtained using (retrospective) conventional measurements. The aortic valve calcium score in the derivation cohort was measured at computed tomography (CT) images to further evaluate the AS severity, using Aquarius iNtuition Edition 4.7. A high calcium score was defined as an Agatston score >1200 in women or >2000 in men, respectively.^1,11^

The likelihood of HFpEF was determined by calculating H_2_FpEF and Heart Failure Association Pre-test assessment, Echocardiography & natriuretic peptide, Functional testing, Final aetiology (HFA-pEFF) scores according to the guidelines, and the components of the score can be found in the supplementary methods.^12,13^ A cut-off of 6 or higher for the H_2_FpEF score and 5 or higher for the HFA-PEFF score was considered as a high likelihood of HFpEF.

The primary endpoints were (1) cardiovascular mortality and (2) HF hospitalizations during 1-year and 5-year follow-up (according to the VARC-3 criteria).^14^ Secondary endpoints included 1-year and 5-year: (1) all-cause mortality, (2) stroke, (3) myocardial infarction (MI), (4) symptom improvement (defined as NYHA class I at 12 months or an improvement of ≥1 NYHA class at 12 months, compared to baseline (in cases where 12-month symptomatic follow-up data was unavailable, symptomatic data from either the 6-month or 30-day follow-up was used)), and (5) a combined endpoint of the adverse events and lack of symptom improvement (only at 1-year follow-up).

### Statistical analysis

Categorical variables were compared using either Chi-square or Fisher’s exact test and presented as n (%). Continuous variables were reported as mean ± standard deviation (SD) for normally distributed variables or median [interquartile range (IQR)] in case of non-normally distributed variables. Continuous variables were compared using *T*-tests or Mann–Whitney tests, as appropriate. A two-sided *P*-value of <.05 was considered statistically significant.

For adequate phenotyping of patients, a cluster analysis based on echocardiographic parameters was performed in the derivation cohort, consisting of patients treated between 2009 and 2020 (*Graphical Abstract*). First, all missing echocardiographic parameters were multiply imputed when the proportion of missing values was less than 90%, using the *mice* package. A sensitivity analysis was conducted using varying thresholds for missing data, ranging from 95% to 50% missing data. The first imputed dataset was taken for subsequent analysis. All parameters were scaled before the analysis. Next, a principal component analysis (PCA) was conducted, with the number of dimensions selected based on the eigenvalue >1. With these principal components, K-means clustering was performed. The optimal number of clusters was determined using the *NbClust* function in R, which evaluates multiple clustering indices.^15^ Least absolute shrinkage and selection operator (LASSO) variable selection was performed to identify the parameters most strongly associated with each cluster. To validate the identified clusters, a similar analysis was performed in a more recent cohort of patients treated in the same centre between 2020 and 2024. In addition, the results were externally validated, as depicted in Supplementary Figure S1. In brief, from the LASSO-derived parameters of the derivation cohort, the most important and available parameters were selected, and centroids were determined. After scaling and standardizing, patients in the external cohort were assigned to the clusters from the derivation cohort based on the shortest distance (Euclidean distance) to the original centroids.^16^

To account for the competing risk of (all-cause) mortality, cumulative incidence curves were generated to evaluate time to cardiovascular mortality and heart failure hospitalizations, and differences between groups were assessed using Gray’s test. All statistical analyses were conducted using R-Studio software (version 4.3.1).

## Results

In total, 827 patients underwent TAVI between 2009 and 2020, of which 199 patients were excluded due to having either NYHA I *n* = 27, or missing parameters (missing NYHA *n* = 1, missing LVEF *n* = 4, missing SVI *n* = 156 and missing AV mean *n* = 38 (or a combination), yielding 628 patients eligible for analysis (mean age 79.1 years (±7.4), 52.1% female, and EuroSCORE II of 4.5%) (*Figure 1*).

At baseline, 446/628 (71%) patients had an LVEF ≥50%. Of these 446 patients, 248 (56%) had a low gradient (LG-AS). In turn, of those, 216 (87%, 35% of total) had an SVI <35 ml/min^2^, thus being identified as the LF-LG pEF group. Baseline characteristics and division of all aortic stenosis flow types can be found in Supplementary Tables S1 and S2 and Supplementary Figure S2.

PCA revealed that the number of dimensions was 50 with an eigenvalue of 1.57 and a cumulative variance of 80.3%, and the optimal number of clusters was *n* = 2. The K-means clustering revealed 2 clusters: Cluster 1 (*n* = 77) and Cluster 2 (*n* = 139).

At baseline, patients in Cluster 1 were older (80.3 (7.35) vs. 78.0 (6.99), *P* = .026), more often had a medical history of AF (56 (72.7%) vs. 24 (17.3%), *P* < .001) and a medical history of HF (27 (35.1%) vs. 21 (15.1%), *P* = .001) (*Table 1*). Cluster 1 patients had higher levels of NT-proBNP and creatinine, lower eGFR levels, and they more often used mineralocorticoid receptor antagonist (MRA), vitamin-K antagonists (VKA), and non-vitamin K antagonist oral anticoagulant (NOAC), while patients in cluster 2 more often used aspirin. Patients in cluster 1 had lower AVA, lower LVEF, lower SVI, lower LA reservoir and LV global longitudinal strain, higher LAVI, elevated PASP, higher E’ Septal, higher E’ Lateral, shorter left ventricular ejection time (LVET), lower tricuspid annular plane systolic excursion (TAPSE), worse right ventricular fractional area change (RV FAC), and lower-systolic excursion velocity of the tricuspid annulus (RV S’ Velocity) (*Table 2*). All patients had high CT Calcium scores, and there was no difference in CT calcium score between both clusters (2005 [1474;2904] versus 2344 [1640;3364], *P* = .397). Patients in cluster 1 had higher numerical HFpEF scores, and more often had a high HFpEF score (H_2_FpEF ≥6 and HFA-pEFF ≥5), all *P* < .001. After combining both scores, 29/77 (37.7%) patients in cluster 1 had high scores when both measures were combined, compared (6/139, 4.3%) in cluster 2 (*P* < .001).

**Table 1:** Baseline characteristics clusters

	Cluster 1	Cluster 2	
	N = 77	N = 139	*P*-value
Demographics			
Age	80.3 (7.35)	78.0 (6.99)	.**026**
Sex (female)	43 (55.8%)	82 (59.0%)	.760
Body mass index	27.9 (4.72)	28.8 (6.53)	.277
New York Heart Association III/IV	71 (92.2%)	118 (84.9%)	.179
EuroSCORE II (Log-)	4.79 (4.25)	3.30 (2.39)	.065
Medical history			
Diabetes	25 (32.5%)	41 (29.5%)	.764
Hypertension	53 (68.8%)	82 (59.0%)	.199
Chronic obstructive pulmonary disease	23 (29.9%)	44 (31.7%)	.906
Atrial fibrillation	56 (72.7%)	24 (17.3%)	**<**.**001**
Coronary artery disease	47 (61.0%)	82 (59.0%)	.882
Myocardial infarction	8 (10.4%)	19 (13.7%)	.629
Percutaneous coronary intervention	34 (44.2%)	57 (41.0%)	.760
Cerebrovascular accident	9 (11.7%)	26 (18.7%)	.251
Prev. Card. Surg.	20 (26.3%)	29 (21.8%)	.568
Coronary artery bypass graft	15 (19.5%)	29 (20.9%)	.948
Peripheral vascular disease	15 (19.5%)	22 (15.8%)	.621
Heart failure	27 (35.1%)	21 (15.1%)	.**001**
Pulmonary hypertension	2 (2.60%)	2 (1.44%)	.618
Porcelain aorta	4 (5.19%)	14 (10.1%)	.325
AV block	17 (22.1%)	23 (16.5%)	.413
Left bundle branch block	3 (3.95%)	4 (2.88%)	.700
RBBB	6 (7.89%)	7 (5.04%)	.390
Pacemaker	9 (11.7%)	9 (6.47%)	.284
Active malignancy	5 (6.49%)	12 (8.76%)	.745
Laboratory values			
Sodium (mmol/L)	139 (4.19)	139 (3.57)	.391
Potassium (mmol/L)	4.36 (0.45)	4.33 (0.46)	.636
Urea (mmol/L)	9.26 (4.11)	8.17 (3.60)	.054
Haemoglobin (mmol/L)	8.10 (1.06)	7.89 (1.00)	.162
NT-proBNP (ng/L)	2369 (2798)	1007 (1971)	**<**.**001**
Logarithmically transformed N-terminal pro-B-type natriuretic peptide	7.33 (0.94)	6.28 (1.04)	**<**.**001**
Creatinine (µmol/L)	105 (41.2)	94.6 (28.4)	.**050**
Estimated glomerular filtration rate (mL/min/1.73 m^2^)	57.1 (16.7)	61.8 (18.0)	.054
Medication use			
Betablockers	59 (76.6%)	88 (63.3%)	.063
Calcium antagonists	21 (27.3%)	43 (30.9%)	.683
Angiotensin-converting enzyme inhibitor/angiotensin receptor blocker	49 (63.6%)	77 (55.4%)	.302
Mineralocorticoid receptor antagonist	13 (16.9%)	8 (5.76%)	.**016**
Statins	41 (53.2%)	86 (61.9%)	.276
Loop diuretics	41 (53.2%)	46 (33.6%)	.**008**
Other diuretics	13 (17.6%)	25 (19.2%)	.915
Insulin	9 (11.7%)	19 (13.7%)	.839
Metformin	15 (19.5%)	23 (16.5%)	.722
Antidiabetics (other)	9 (11.7%)	13 (9.35%)	.757
Aspirin	22 (28.6%)	84 (60.4%)	**<**.**001**
Vitamin-K antagonist	46 (59.7%)	17 (12.2%)	**<**.**001**
Immunosuppressive	7 (9.46%)	16 (12.7%)	.643
Non-vitamin K antagonist direct oral anticoagulant	12 (15.6%)	8 (5.80%)	.**034**
Clopidogrel (P2y12)	19 (24.7%)	36 (26.1%)	.949
CT calcium score			
Agatston Score	2005 [1474;2904]	2344 [1640;3364]	.397
High score (>1200 (F) or >2000 (M))	17 (63.0%)	52 (78.8%)	.186
HFpEF scores			
H_2_FpEF (numerical)	6.00 [4.00;7.00]	3.00 [2.00;4.00]	**<**.**001**
High (≥6)	40 (51.9%)	18 (12.9%)	**<**.**001**
HFA-pEFF (numerical)	5.00 [4.00;6.00]	4.00 [3.00;5.00]	**<**.**001**
High (≥5)	55 (71.4%)	62 (44.6%)	**<**.**001**
Combined			**<**.**001**
Both low	13 (16.9%)	66 (47.5%)	
High H_2_FpEF, Low HFA-pEFF	9 (11.7%)	11 (7.91%)	
Low H_2_FpEF, High HFA-pEFF	24 (31.2%)	55 (39.6%)	
**Both high**	**31 (40.3%)**	**7 (5.04%)**	

Values are shown as n (%) for categorical variables and mean ± (SD) or median [IQR] for continuous variables.

A *P* value of <.05 was considered as statistically signiﬁcant.

**Table 2:** Baseline echocardiographic parameters clusters

	Cluster 1	Cluster 2	
	N = 77	N = 139	*P* value
Aortic valve area	0.79 (0.24)	0.87 (0.23)	.**023**
Aortic mean gradient	27.9 (7.53)	29.5 (7.38)	.132
Left ventricular ejection fraction	60.4 (6.77)	62.9 (6.32)	.**009**
Stroke volume index	22.0 (5.52)	25.4 (5.22)	**<**.**001**
Transvalvular flow rate	142 (38.1)	151 (39.9)	.113
Flow rate			.836
Low-flow rate	67 (90.5%)	122 (92.4%)	
Normal flow rate	7 (9.5%)	10 (7.6%)	
Left atrial reservoir strain	9.40 (6.2)	25.5 (8.0)	**<**.**001**
Left ventricular global longitudinal strain	−13.99 (3.4)	−17.68 (3.6)	**<**.**001**
Left ventricular mass index	83.4 (19.2)	84.1 (20.5)	.822
Left atrial volume index	47.7 (12.5)	30.3 (7.00)	**<**.**001**
Pulmonary artery systolic pressure	43.4 (13.2)	34.7 (13.2)	**<**.**001**
E	108 (22.7)	80.7 (20.4)	**<**.**001**
e’ septal	6.97 (2.07)	5.62 (1.81)	.**001**
e’ lateral	9.00 (2.23)	6.82 (2.04)	**<**.**001**
E/e’	14.8 (3.13)	13.1 (4.76)	.072
TR V Max	2.99 (0.55)	2.66 (0.60)	.**001**
TR Peak gradient	36.9 (12.7)	29.8 (13.1)	.**001**
MR jet area (A4C)	5.43 (3.79)	4.41 (3.15)	.254
MR jet/LA area ratio (A4C)	23.6 (13.3)	23.6 (15.8)	.997
Mitral regurgitation vena contracta width	0.40 (0.17)	0.38 (0.11)	.666
Mitral annulus diameter (A4C)	32.0 (3.84)	28.0 (3.59)	**<**.**001**
Left ventricular outflow tract	19.0 (2.23)	18.8 (2.34)	.731
Interventricular septal thickness in diastole	11.8 (2.25)	11.4 (2.71)	.319
Left ventricular posterior wall thickness in diastole	11.1 (2.60)	10.5 (1.63)	.107
Relative wall thickness	0.56 (0.16)	0.51 (0.11)	.088
Left ventricular ejection time	303 (35.2)	323 (37.9)	**<**.**001**
Tricuspid annular plane systolic excursion	19.1 (6.03)	22.6 (4.41)	.**001**
Tricuspid annular plane systolic excursion/pulmonary artery systolic pressure ratio	0.53 (0.45)	0.86 (0.58)	.**024**
Right ventricular fractional area change (%)	34.5 (12.6)	40.7 (11.8)	.**007**
Fractional area change/pulmonary artery systolic pressure ratio	0.81 (0.39)	1.35 (0.82)	**<**.**001**
RV S’ velocity	8.76 (2.59)	11.0 (3.66)	.**003**
**S'/pulmonary artery systolic pressure ratio**	**0.22 (0.08)**	**0.34 (0.15)**	.002

Values are shown as n (%) for categorical variables and mean ± (SD) or median [IQR] for continuous variables.

A *P* value of <.05 was considered as statistically signiﬁcant.

E, early mitral inflow velocity; E/e′, ratio of early mitral inflow to early diastolic mitral annular velocity; e′ lateral, lateral mitral annular early diastolic velocity; e′ septal, septal mitral annular early diastolic velocity; LA, left atrial; LV, left ventricular; TR P Max, tricuspid regurgitation peak pressure gradient; TR V Max, tricuspid regurgitation peak velocity; RV S’ velocity, tissue Doppler-derived systolic velocity of the tricuspid annulus; S'/PASP ratio, ratio of RV S’ velocity to pulmonary artery systolic pressure.

Sensitivity analysis using different thresholds for allowed missing percentage of included variables (5%–95%) revealed a similar trend, identifying two clusters, including one with high H_2_FpEF scores and high percentage of AF (Supplementary Table S3).

**Figure 2:**
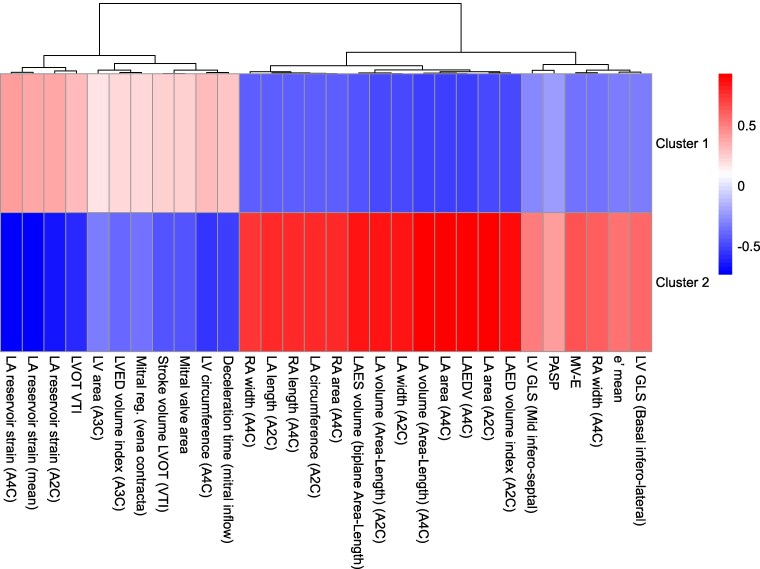
Heatmap least absolute shrinkage and selection operator-derived echo-parameters. Heatmap showing the most important parameters associated with both clusters, derived by least absolute shrinkage and selection operator regression analysis. Numbers are scaled values.

Peri-procedurally, patients in cluster 1 less often had a transfemoral (TF) approach compared to patients in cluster 2 (57 (74.0%) vs. 120 (86.3%), *P* = .039) (Supplementary Table S4). There were no statistically significant differences regarding clinical presentation for TAVI, expansion type, femoral size, implanted valve size, or anaesthesia type between both groups. LASSO logistic regression analysis revealed 30 echocardiographic variables as most associated with the two clusters (*Figure 2*, Supplementary Table S5). Patients in cluster 1 had larger left atrial dimensions characterized by larger LAEDV, LA volume, width, circumference, and right atrial dimensions, as shown by RA length, area, and width. Also, patients in cluster 1 had lower LA reservoir strain (in all views). Also, PASP was elevated in cluster 1 compared to cluster 2, with higher rates of MV E. Patients in cluster 2, on the other hand, have larger left ventricles (LV area and circumference), close to normal LA and RA dimensions, larger mitral valve (MV), and longer MV deceleration time.

**Figure 3:**
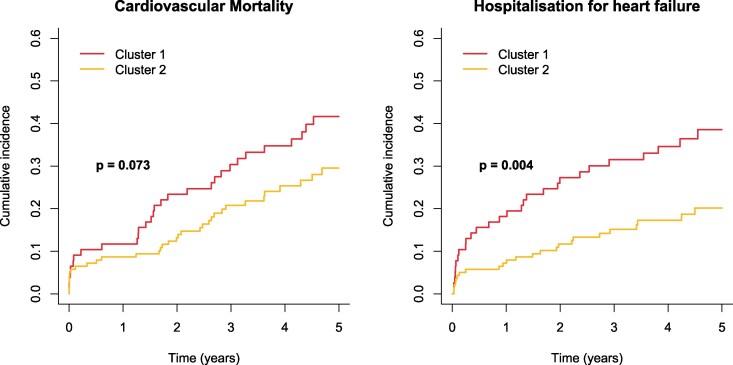
Cumulative incidence of events in clusters. Cumulative incidence curves for both clusters in heart failure hospitalizations and cardiovascular mortality, during 5-year follow-up, with all-cause mortality as a competing event.

**Figure 4:**
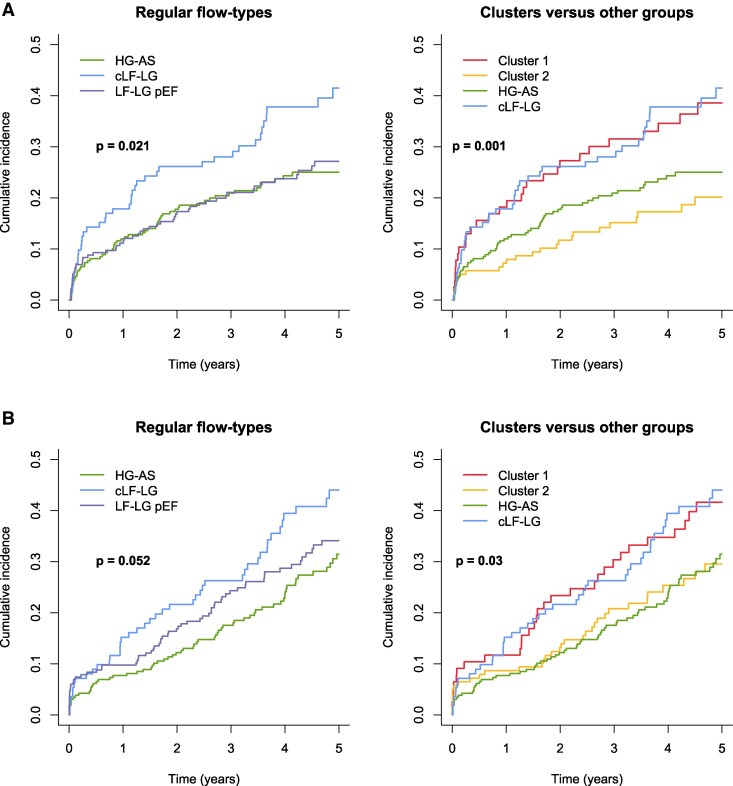
Cumulative incidence of flow types versus clusters. Cumulative incidence curves for both clusters and other flow types for (A) cardiovascular mortality and (B) Heart failure hospitalization, during 5-year follow-up, with all-cause mortality as a competing event.

There were no statistically significant differences within both clusters with respect to peri-procedural complications (Supplementary Table S6). At 1-year follow-up, there was no statistically significant difference in rates of cardiovascular mortality between both clusters, also accounting for stroke, lack of symptom improvement, and MI. Still, patients in Cluster 1 had higher rates of HF hospitalizations (14 (18.2%) vs. 10 (7.19%), *P* = .025) (*Table 3*). This higher rate of HF hospitalizations was also seen at 5-year follow-up (28 (36.4%) vs. 24 (17.3%), *P* = .003). Patients in Cluster 1 had higher rates of cardiovascular mortality at 5 years (30 (39.0%) vs. 34 (24.5%), *P* = .038). Cumulative incidence curves showed that patients in Cluster 1 had higher rates of HF hospitalizations (*P* = .004) and a trend towards higher cardiovascular mortality (*P* = .073) (*Figure 3*). When comparing outcomes of the two clusters with other flow types, patients in cluster 1 had worse outcomes, comparable to the classical LF-LG (cLF-LG) patients, while patients in cluster 2 exhibited outcomes similar to high gradient (HG) patients (*Figure 4*). Outcomes of all groups can be found in Supplementary Table S7. The course of (log-)NT-proBNP after the procedure at 30 days, 6 and 12 months is shown in Supplementary Figure S3, revealing higher NT-proBNP levels in cluster 1 compared to cluster 2, at all time-points.

**Table 3:** Outcomes clusters

	Cluster 1	Cluster 2	*P-value*
	N = 77	N = 139	
**1-year**			
Cardiovascular mortality	9 (11.7%)	10 (7.19%)	.386
All-cause mortality	11 (14.3%)	17 (12.2%)	.826
Heart failure hospitalisation	14 (18.2%)	10 (7.19%)	.**025**
Stroke	4 (5.19%)	16 (11.5%)	.197
Myocardial infarction	0 (0.00%)	3 (2.16%)	.554
Lack of symptom improvement	20 (26.0%)	35 (25.2%)	1.000
Combined endpoint^a^	12 (21.1%)	17 (15.6%)	.507
**5-year**			
Cardiovascular mortality	30 (39.0%)	34 (24.5%)	.**038**
All-cause mortality	43 (55.8%)	60 (43.2%)	.100
Heart failure hospitalization	28 (36.4%)	24 (17.3%)	.**003**
Stroke	6 (7.79%)	24 (17.3%)	.085
Myocardial infarction	2 (2.60%)	4 (2.88%)	1.000

Values are n (%). A *P* value of <.05 was considered as statistically significant.

^a^Combined endpoint: composite endpoint of cardiovascular mortality, stroke, or heart failure hospitalizations, together with lack of symptom improvement.

To validate the identified clusters, the same clustering technique was performed internally in another patient cohort in the same centre, as can be found in Supplementary Tables S8, S9, and S10. In brief, 405 patients were eligible for analysis, of which 111 had a LF-LG pEF flow-type. Despite baseline differences between the cohorts in the two time periods, two distinct clusters were again identified. One of these clusters included patients with high HFpEF scores; they were less often female, more often had AF, and higher NT-proBNP levels.

In addition, external validation was performed in an independent external cohort of 173 patients from Munich, Germany (mean age 82.4 years old, 55.5% female, all LF-LG pEF) (Supplementary Table S11). After assigning the two clusters to the two cohorts (based on shortest Euclidian distance), 93 patients (53% of the patients) were assigned to cluster 1 and 80 (47%) to cluster 2. Patients in cluster 1 again exhibited higher HFpEF scores, higher incidence of AF, impaired LA reservoir strain and GLS, and higher NT-proBNP values, closely mirroring the derivation results. This confirms the reproducibility and generalizability of the clustering approach across centres and countries.

## Discussion

The main findings of this retrospective analysis of a real-world cohort of patients undergoing TAVI were:

1. A substantial subset (35%) of patients undergoing TAVI have a paradoxical LF-LG pEF phenotype.
2. Based on unsupervised AI-echocardiographic clustering, only one-third of these LF-LG pEF patients have a HFpEF phenotype, further characterized by AF, large LA and RA diameters, and impaired LA reservoir strain and LV GLS.
3. The results and characterizations of this HFpEF phenotype were validated in an internal and independent external cohort, yielding very similar results, showing high reproducibility and generalizability.
4. Patients with this HFpEF/AF phenotype have a higher risk of 5-year HF hospitalizations and outcomes worse than that of cLF-LG patients, while patients without this HFpEF/AF phenotype have similar outcomes to HG patients. Patients with a HFpEF phenotype could potentially benefit from medical therapy.

Over half of the patients included in this cohort of TAVI patients had a low gradient, of which half had a pEF status, in line with previous reports.^3,17^ While these patients have a low gradient and a paradoxical pEF status, most of them had a high degree of calcifications (measured by CT calcium scores), supporting the diagnosis of a (severe) aortic stenosis. They were also highly symptomatic at baseline (high NYHA class).^1^

Based on AI-assessed echocardiographic parameters, two clusters within LF-LG pEF patients were identified. Cluster 1 had notably higher HFpEF scores, consistent with a HFpEF phenotype. Further, cluster 1 also demonstrated a high prevalence of a history of AF. Other characteristics that were more prevalent in cluster 1 were a history of HF, together with high NT-proBNP levels and medications related to this HF(pEF) and AF. These findings of two distinct clusters were also found in the internal and external validation cohorts. It is known that patients with a LF-LG pEF have multiple comorbidities and similarities with HFpEF, such as the same abnormal functional parameters.^5,6,18^ Based on our results, however, not all patients with this LF-LG pEF type have HFpEF or AF. Echocardiographic parameters most strongly associated with cluster 1 reflect general LA dysfunction, by larger dimensions of the left atrium (LA) (elevated LAEDV(i) and LA area), and a reduced LA reservoir strain, strongly related to HFpEF.^19^ Here again, it should be noted that there was a high prevalence of a history of AF in cluster 1, sharing similar pathophysiologic mechanisms with HFpEF.^20^ In addition, right atrial (RA) dimensions were enlarged in cluster 1 patients, together with an elevated PASP. The findings of a low TAPSE, RV FAC%, and RV S’ velocity in cluster 1 further indicate that right ventricular systolic dysfunction is accompanied by other characteristics that are typical for patients with HFpEF.^21^ Furthermore, a reduced LV longitudinal strain was found in cluster 1. The SVI was low in both clusters. While this discordant grading can be the consequence of HFpEF in cluster 1, in cluster 2 it can be a result of other conditions, such as mitral regurgitation (reflected by the large MV area) or, alternatively, mitral stenosis (slightly elevated deceleration time).^7^ This low-flow, low gradient status in cluster 2 could have resulted from incorrect echocardiographic measurements, which is supported by the finding that only 63% of the patients in cluster 1 had a high Agatston score. It could be hypothesized that these patients were misclassified as severe aortic stenosis and should therefore prompt further investigation, for example, by dobutamine stress echocardiography (DSE) or invasive AVA and gradient measurements under infusion of nitroprusside.^22^

The HFpEF/AF phenotype cluster (cluster 1) had worse outcomes compared to patients in cluster 2, both at 1- and 5-year follow-up. While patients cluster 1 more often had a non-TF approach, there were no differences in peri-procedural complications, including no differences in PVL or aortic regurgitation. Previous reports showed that patients with a high HFA-pEFF and H_2_FpEF score had worse outcomes, but data regarding low gradient patients remained lacking.^23–27^ When comparing patients in the two clusters with other flow types, cluster 1 showed even worse outcomes than cLF-LG patients. This cLF-LG group often represents patients with heart failure with a reduced ejection fraction (HFrEF) and existing literature showed worse outcomes in HFrEF patients undergoing TAVI when compared to HFpEF patients, although these studies did not include HFpEF scores or invasive measurements to diagnose pure HFpEF.^28^ It can thus be hypothesized that HFpEF/AF patients with a low gradient status reflect an advanced disease and have a higher risk of adverse events, similar to HFrEF patients. This is supported by the elevated NT-proBNP levels in cluster 1. As previously shown, high NT-proBNP levels at baseline predict adverse events after TAVI, and these stable high levels suggest the inability of TAVI to fully treat and reverse the cardiac damage.^29^ Patients with a LF-LG, high HFpEF scores, and high NT-proBNP levels at the time of TAVI should thus be considered as a high-risk patient population. Cluster 2 patients, without this HFpEF/AF phenotype, on the other hand, have a prognosis similar to HG patients.

### Implications

To the best of our knowledge, we are the first to in-depth phenotype LF-LG pEF patients undergoing TAVI by an unsupervised, AI echocardiography–based cluster analysis, and associate this with HFpEF. We showed that only a subset, one-third of these patients, have a clear HFpEF/AF phenotype. These findings were confirmed not only in an internal, more recently treated subset of patients, but also in an independent, international, external cohort. As the HFpEF/AF phenotype patients have worse outcomes, they likely benefit from guideline-directed medical therapies for patients with HFpEF, including SGLT-2 inhibitors and the MRA finerenone.^30^ Moreover, we demonstrated better outcomes of patients with a non-HFpEF/AF LF-LG phenotype than previous studies, comparable to outcomes of patients with a HG AS. This emphasizes the necessity of detailed echocardiographic and invasive assessment of aortic stenosis and HFpEF to risk stratify and phenotype patients properly.

### Limitations

This study has several limitations. Its retrospective observational design resulted in non-standardized (symptomatic) outcomes assessed by different clinicians. Patient-reported outcomes were not included due to unavailability; future research should incorporate measures such as the KCCQ or SF-12 to evaluate their alignment with clinician-reported outcomes. Secondly, we did not have access to data on DSE, which is part of the diagnostic work-up in cases of classical LF-LG grading, but not in paradoxical LF-LG patients. Thus, we are not reporting it here in the paradoxical LF-LG group. Lastly, while validated in large cohorts, both HFpEF scores were not validated in an aortic stenosis or TAVI-specific patient population, and previous literature showed varying diagnostic performance.^25,26,31^ Invasive haemodynamic measurements during right heart catheterization of all these patients should be performed in future research, possibly with the use of nitroprusside infusion.

## Conclusion

One third of the patients undergoing TAVI have a LF-LG pEF aortic stenosis. Unsupervised clustering based on more than 300 AI-assessed echocardiographic parameters identified that only one-third of these patients had a typical HFpEF/AF phenotype, which was internal and (independently) externally validated. These patients have a poor prognosis at both 1- and 5-year follow-up, similar to cLF-LG patients, and could potentially benefit from guideline-directed medical therapies for patients with HFpEF, including SGLT-2 inhibitors and the MRA. This emphasizes the need for more detailed phenotyping and evaluation of these patients, as well as further research on more adjunctive treatment.

## Perspectives

- **What is Known?** Patients with a low-flow, low-gradient aortic stenosis have worse outcomes after TAVI.
- **What is New?** Not all patients with a LF-LG pEF aortic stenosis have a HFpEF/AF phenotype, but when they do, they have worse outcomes after TAVI, comparable to cLF-LG.
- **What is Next**? Larger, multi-centre studies are necessary to validate these results, together with the inclusion of invasive haemodynamic measurements.

## Supplementary Material

xvag146_Supplementary_Data

## Declarations

### Data Availability

No data were generated or analyzed for this manuscript.

### Pre-registered Clinical Trial Number

None supplied.
