# Supplementary material for: Phenotyping patients with a low-flow, low-gradient aortic stenosis and a preserved ejection fraction undergoing transcatheter aortic valve implantation
Source: ESC Heart Fail. 2026 May 31;13(4):xvag146. doi: 10.1093/eschf/xvag146 (PMC13482987; doi:10.1093/eschf/xvag146)
Supplement: Supplementary file 1 [file xvag146_supplementary_data.zip › LF-LG-pEF_Supplementary Material_Final_Version_22_Dec.docx]

Supplementary Material

[**Supplementary Methods**	2](#_Toc215149459)

[**Supplementary Table 1:** Baseline characteristics	4](#_Toc215149460)

[**Supplementary Table 2:** Baseline and H_2_FpEF-score in total cohort	6](#_Toc215149461)

[**Supplementary Table 3:** Clusters and H_2_FPEF score and percentage of AF for different missing numbers thresholds.	7](#_Toc215149462)

[**Supplementary Table 4:** Peri-procedural characteristics	8](#_Toc215149463)

[**Supplementary Table 6:** Peri-procedural complications and outcomes	10](#_Toc215149464)

[**Supplementary Table 7:** Outcomes complete cohort	11](#_Toc215149465)

[**Supplementary Table 8:** Flow-types validation and derivation cohort	12](#_Toc215149466)

[**Supplementary Table 10:** Clusters internal validation cohort	14](#_Toc215149467)

[**Supplementary Table 11:** Baseline and clusters external validation cohort	15](#_Toc215149468)

[**Supplementary Figure 1: Me**thods external validation	16](#_Toc215149469)

[**Supplementary Figure 2:** Flow-diagram Inclusion, regular flow-types	17](#_Toc215149470)

[**Supplementary Figure 3:** Course of NT-proBNP during 1-year follow-up	18](#_Toc215149471)

### **Supplementary Methods**

The flow types of AS were defined as follows: high-gradient AS (HG-AS) was defined as AVA ≤1.0 cm² and AV MG ≥40 mmHg. Classical low-flow, low-gradient AS (cLF LG-AS) was defined as an AVA <1.0 cm², left ventricular ejection fraction (LVEF) <50%, AV MG <40 mmHg, and SVI <35 ml/m². LF-LG pEF ((LVEF ≥50%) was defined as an AVA <1.0 cm², AV MG <40 mmHg, and SVI <35 ml/m², while normal-flow, low-gradient AS (NF LG-AS) was defined as an AVA <1.0 cm², LVEF ≥50%, MG <40 mmHg, and SVI >35 ml/m²(1,2).

The H_2_FpEF score comprises of: body mass index (BMI) > 30 kg/m² (2 points, heavy), the presence of treated or untreated hypertension (1 point, hypertension), atrial fibrillation (either persistent or paroxysmal) (3 points), pulmonary hypertension with an estimated pulmonary artery systolic pressure (PASP) gr>35 mmHg (1 point), age over 60 years (1 point, elderly), and an E/e’ ratio exceeding 9, indicative of elevated filling pressure (1 point)(3).

The HFA-PEFF score integrates functional, morphological, and biomarker domains to classify heart failure phenotypes(4). Within each domain, parameters are categorized as major or minor criteria, corresponding to 2 and 1 point(s) respectively. In the functional domain, major criteria include a septal e’ <7 cm/s, lateral e’<10 cm/s, E/e’ ratio greater than 15, or a tricuspid regurgitation velocity exceeding 2.8 m/s (PASP >35 mmHg) (2 point) is considered, or E/e’ ratio of 9-14 or GLS of<16% (1 point) In the morphological domain, a left atrial volume index greater than 34 mL/m² or a left ventricular mass index exceeding 149 g/m² in men or 122 g/m² in women with a RWT >0.42 (2 points), or a left atrial volume index between 29 and 34 mL/m², or a left ventricular mass index exceeding 115 g/m² in men or 95 g/m² in women or a RWT >0.42, or a LV wall thickness >12 mm (1 point) is assessed. In the biomarker domain, NT-proBNP levels above 220 pg/mL or BNP levels exceeding 80 pg/mL (2 point), NT-proBNP of 125-220, or BNP 35-80 (1 point) when in sinus rhythm, or NT-proBNP >660 or BNP >240 (2 points) or NT-proBNP 365-660 or BNP 105-250 (1 point) in atrial fibrillation are evaluated. A cut-off of score above 5 was considered as a high likelihood of HFpEF.

1.	Nakase M., Okuno T., Tomii D., et al. Prognostic impact of cardiac damage staging classification in each aortic stenosis subtype undergoing TAVI. EuroIntervention 2023;19(10):E865–74. Doi: 10.4244/EIJ-D-23-00590.

2.	Nakase M., Tomii D., Maznyczka A., et al. Sex-Specific Differences in Upstream Cardiac Damage in Patients With Aortic Stenosis Undergoing TAVR. JACC Cardiovasc Interv 2024;17(10):1252–64. Doi: 10.1016/j.jcin.2024.03.031.

3.	Reddy YNV., Carter RE., Obokata M., Redfield MM., Borlaug BA. A simple, evidence-based approach to help guide diagnosis of heart failure with preserved ejection fraction. Circulation 2018;138(9):861–70. Doi: 10.1161/CIRCULATIONAHA.118.034646.

4.	Pieske B., Tschöpe C., De Boer RA., et al. How to diagnose heart failure with preserved ejection fraction: The HFA-PEFF diagnostic algorithm: A consensus recommendation from the Heart Failure Association (HFA) of the European Society of Cardiology (ESC). Eur Heart J 2019;40(40):3297–317. Doi: 10.1093/EURHEARTJ/EHZ641,.

### **Supplementary Table 1:** Baseline characteristics

|  | **HG-AS** | **cLF-LG** | **LF-LG pEF** | **NF-LG** | **p-value** |
| --- | --- | --- | --- | --- | --- |
|  | **N=261** | **N=112** | **N=216** | **N=32** |  |
| Age | 80.5 (6.81) | 77.8 (7.30) | 78.8 (7.19) | 74.8 (10.7) | **<0.001** |
| Sex (female) | 146 (55.9%) | 43 (38.4%) | 125 (57.9%) | 13 (40.6%) | **0.002** |
| BMI | 27.2 (5.05) | 27.6 (6.21) | 28.5 (5.95) | 27.8 (6.01) | 0.106 |
| NYHA III/IV | 208 (79.7%) | 101 (90.2%) | 189 (87.5%) | 28 (87.5%) | **0.032** |
| EuroSCORE II (Log-) | 4.02 (3.06) | 6.89 (6.91) | 3.80 (3.19) | 3.87 (3.27) | **<0.001** |
| **Medical History** | | | | | |
| Diabetes | 73 (28.0%) | 48 (42.9%) | 66 (30.6%) | 9 (28.1%) | **0.038** |
| Hypertension | 138 (52.9%) | 68 (60.7%) | 135 (62.5%) | 15 (46.9%) | 0.092 |
| COPD | 58 (22.2%) | 25 (22.3%) | 67 (31.0%) | 8 (25.0%) | 0.136 |
| AF | 74 (28.4%) | 43 (38.4%) | 80 (37.0%) | 11 (34.4%) | 0.137 |
| CAD | 122 (46.7%) | 81 (72.3%) | 129 (59.7%) | 13 (40.6%) | **<0.001** |
| MI | 38 (14.6%) | 33 (29.5%) | 27 (12.5%) | 6 (18.8%) | **0.001** |
| PCI | 85 (32.6%) | 52 (46.4%) | 91 (42.1%) | 9 (28.1%) | **0.023** |
| CVA | 40 (15.3%) | 23 (20.5%) | 35 (16.2%) | 1 (3.12%) | 0.123 |
| Prev. Card. Surg. | 40 (15.6%) | 35 (31.5%) | 49 (23.4%) | 9 (28.1%) | **0.004** |
| CABG | 32 (12.3%) | 33 (29.5%) | 44 (20.4%) | 9 (28.1%) | **0.001** |
| PVD | 35 (13.4%) | 14 (12.5%) | 37 (17.1%) | 7 (21.9%) | 0.370 |
| HF | 77 (29.5%) | 63 (56.2%) | 48 (22.2%) | 10 (31.2%) | **<0.001** |
| Pulm. hyper. | 3 (1.15%) | 2 (1.79%) | 4 (1.85%) | 1 (3.12%) | 0.568 |
| Porc. AoV | 14 (5.36%) | 5 (4.46%) | 18 (8.33%) | 6 (18.8%) | **0.034** |
| AV block | 40 (15.3%) | 22 (19.6%) | 40 (18.5%) | 8 (25.0%) | 0.463 |
| LBBB | 16 (6.18%) | 18 (16.5%) | 7 (3.26%) | 3 (9.38%) | **<0.001** |
| RBBB | 28 (10.9%) | 18 (16.5%) | 13 (6.05%) | 3 (9.38%) | **0.027** |
| Pacemaker | 17 (6.51%) | 15 (13.4%) | 18 (8.33%) | 6 (18.8%) | **0.038** |
| Active Malignancy | 24 (9.20%) | 14 (12.5%) | 17 (7.94%) | 3 (9.38%) | 0.592 |
| **Laboratory Findings** | | | | | |
| Na | 139 (3.42) | 139 (3.29) | 139 (3.80) | 139 (2.50) | 0.552 |
| K | 4.28 (0.38) | 4.35 (0.46) | 4.34 (0.46) | 4.35 (0.37) | 0.346 |
| Urea | 8.33 (3.95) | 9.63 (4.60) | 8.56 (3.82) | 8.63 (4.69) | 0.045 |
| Hb | 7.84 (1.05) | 7.97 (0.97) | 7.96 (1.03) | 7.37 (1.13) | **0.016** |
| NTpro-BNP (pre) | 3516 (5496) | 4874 (6704) | 1486 (2381) | 1735 (2326) | **<0.001** |
| Log NT-proBNP | 7.38 (1.28) | 7.82 (1.23) | 6.65 (1.12) | 6.76 (1.28) | **<0.001** |
| Creatinine | 97.7 (46.2) | 106 (32.7) | 98.3 (33.8) | 93.8 (25.9) | 0.229 |
| eGFR | 61.2 (18.8) | 57.5 (16.9) | 60.1 (17.7) | 65.8 (20.6) | 0.102 |
| **Medication use at baseline** | | | | | |
| Betablockers | 136 (52.3%) | 79 (70.5%) | 147 (68.1%) | 15 (46.9%) | **<0.001** |
| Calcium Antagonists | 69 (26.5%) | 25 (22.3%) | 64 (29.6%) | 9 (28.1%) | 0.562 |
| ACE/ARB | 140 (53.8%) | 67 (59.8%) | 126 (58.3%) | 16 (50.0%) | 0.556 |
| MRA | 41 (15.8%) | 30 (27.0%) | 21 (9.72%) | 7 (22.6%) | **0.001** |
| Statins | 137 (52.5%) | 73 (65.2%) | 127 (58.8%) | 20 (62.5%) | 0.119 |
| Loop diuretics | 102 (39.4%) | 65 (60.7%) | 87 (40.7%) | 12 (38.7%) | **0.001** |
| Other Diuretics | 42 (16.7%) | 8 (7.84%) | 38 (18.6%) | 5 (16.7%) | 0.077 |
| Insulin | 26 (10.0%) | 22 (19.6%) | 28 (13.0%) | 5 (15.6%) | 0.084 |
| Metformin | 39 (15.0%) | 29 (25.9%) | 38 (17.6%) | 9 (28.1%) | **0.041** |
| Anti-diabetics (other) | 22 (8.49%) | 22 (19.6%) | 22 (10.2%) | 3 (9.38%) | **0.023** |
| Aspirin | 127 (48.8%) | 51 (45.5%) | 106 (49.1%) | 14 (43.8%) | 0.879 |
| VKA | 60 (23.1%) | 36 (32.1%) | 63 (29.2%) | 13 (40.6%) | 0.079 |
| Immunosuppressive | 17 (7.20%) | 10 (10.2%) | 23 (11.5%) | 3 (9.68%) | 0.448 |
| NOAC | 17 (6.54%) | 16 (14.3%) | 20 (9.30%) | 1 (3.12%) | 0.078 |
| Clopidogrel (P2y12) | 69 (26.5%) | 34 (30.4%) | 55 (25.6%) | 9 (28.1%) | 0.823 |
| **Echocardiographic parameters** | | | | | |
| AVA | 0.70 (0.21) | 0.79 (0.38) | 0.84 (0.24) | 0.93 (0.27) | **<0.001** |
| AVMean | 52.8 (11.3) | 27.2 (7.49) | 28.9 (7.46) | 30.8 (7.05) | **<0.001** |
| LVEF | 57.6 (11.6) | 38.1 (7.48) | 62.0 (6.58) | 62.5 (7.30) | **<0.001** |
| SVI | 28.8 (8.01) | 23.3 (5.36) | 24.2 (5.55) | 39.5 (4.00) | **<0.001** |
| Transvalvular Flow Rate | 168 (49.2) | 148 (42.1) | 147 (39.4) | 244 (51.3) | **<0.001** |
| Flow Rate: |  |  |  |  | **<0.001** |
| Low-FR | 161 (72.9%) | 95 (89.6%) | 189 (91.7%) | 7 (21.9%) |  |
| Normal-FR | 60 (27.1%) | 11 (10.4%) | 17 (8.25%) | 25 (78.1%) |  |
| LARes | 19.3 (10.5) | 13.7 (8.48) | 19.3 (10.7) | 21.0 (11.6) | **<0.001** |
| LVGls | -15.62 (4.49) | -10.16 (3.32) | -16.38 (3.96) | -16.26 (4.57) | **<0.001** |
| LVMi | 102 (28.4) | 110 (22.6) | 83.9 (20.0) | 106 (30.7) | **<0.001** |
| LAVI | 39.7 (12.0) | 41.4 (11.8) | 36.0 (12.2) | 38.2 (15.2) | **0.005** |
| PASP | 49.5 (25.3) | 41.5 (16.2) | 38.1 (13.8) | 38.7 (14.1) | **<0.001** |
| E | 95.1 (30.0) | 96.2 (35.4) | 90.3 (24.9) | 99.1 (26.0) | 0.265 |
| e' septal | 5.19 (1.71) | 4.83 (1.78) | 6.07 (2.00) | 6.06 (1.68) | **<0.001** |
| e' lateral | 7.01 (2.36) | 7.47 (2.40) | 7.57 (2.34) | 7.78 (2.84) | 0.217 |
| E/e’ | 15.0 (6.04) | 16.3 (7.03) | 13.5 (4.50) | 14.7 (5.21) | 0.076 |
| TR V Max | 3.19 (0.94) | 2.89 (0.64) | 2.80 (0.60) | 2.90 (0.66) | **<0.001** |
| TR Peak gradient | 44.2 (25.8) | 35.1 (15.8) | 32.7 (13.3) | 35.3 (15.1) | **<0.001** |
| TR vena contracta width | 1.22 (1.75) | 1.28 (1.60) | 2.14 (2.32) | 1.17 (1.72) | 0.123 |
| MR jet area | 5.09 (3.35) | 5.14 (3.52) | 4.94 (3.51) | 7.89 (5.09) | 0.345 |
| MR jet/LA area ratio | 24.7 (15.0) | 26.2 (14.5) | 23.6 (14.4) | 51.2 (29.7) | **0.002** |
| MR Vena contracta width | 0.39 (0.13) | 0.44 (0.11) | 0.39 (0.14) | 0.42 (0.11) | 0.619 |
| Mitral annulus diameter | 30.4 (4.55) | 32.3 (3.97) | 29.3 (4.12) | 29.8 (3.86) | **<0.001** |
| LVOT | 18.9 (2.45) | 19.6 (2.44) | 18.9 (2.29) | 19.4 (2.57) | 0.105 |
| IVSd | 11.5 (2.13) | 10.6 (1.98) | 11.3 (2.26) | 10.8 (1.64) | **0.007** |
| LVPWd | 11.3 (2.28) | 11.3 (2.50) | 10.7 (2.05) | 11.3 (2.82) | 0.081 |
| RWT | 0.51 (0.14) | 0.46 (0.13) | 0.53 (0.13) | 0.47 (0.14) | **<0.001** |
| LVET | 320 (32.9) | 308 (38.5) | 315 (38.1) | 318 (42.8) | **0.046** |
| Values are shown as n (%) for categorical variables and mean ± (SD) or median [IQR] for continuous variables.  A P value of <0.05 was considered as statistically signiﬁcant.  ACE/ARB: Angiotensin-Converting Enzyme Inhibitor / Angiotensin Receptor Blocker, Anti-diabetics (other): Other medications for diabetes besides insulin and metformin, AV-Block: Atrioventricular Block, AF: Atrial Fibrillation, BMI: Body Mass Index, CAD: Coronary Artery Disease, CABG: Coronary Artery Bypass Graft, COPD: Chronic Obstructive Pulmonary Disease, CVA: Cerebrovascular Accident, eGFR: Estimated Glomerular Filtration Rate, (Log-)NT-proBNP: (Logarithmically transformed) N-terminal pro-B-type Natriuretic Peptide, MI: Myocardial Infarction, MRA: Mineralocorticoid Receptor Antagonist, NOAC: Non-vitamin K antagonist direct oral anticoagulant, PCI: Percutaneous Coronary Intervention, Pulm. HT: Pulmonary Hypertension, VKA: Vitamin-K Antagonist  AVA: aortic valve area; AV: aortic; E: early mitral inflow velocity; E/e′: ratio of early mitral inflow to early diastolic mitral annular velocity; e′ lateral: lateral mitral annular early diastolic velocity; e′ septal: septal mitral annular early diastolic velocity; IVSd: interventricular septal thickness in diastole; LA: left atrial; LAVI: left atrial volume index; LVEF: left ventricular ejection fraction; LVET: left ventricular ejection time; LV: left ventricular, LVMi: left ventricular mass index; LVOT: left ventricular outflow tract; LVPWd: left ventricular posterior wall thickness in diastole; MR: mitral regurgitation FR: flow rate (normal: ≥250 mL/s); PASP: pulmonary artery systolic pressure; RWT: relative wall thickness; SVI: stroke volume index; TR P Max: tricuspid regurgitation peak pressure gradient; TR V Max: tricuspid regurgitation peak velocity. | | | | | |

### **Supplementary Table 2:** Baseline and H_2_FpEF-score in total cohort

|  | **HG-AS** | **cLF-LG** | **LF-LG pEF** | **NF-LG** | **p-value** |
| --- | --- | --- | --- | --- | --- |
|  | **N=261** | **N=112** | **N=216** | **N=32** |  |
| **H_2_FpEF (numerical)** | 3.00 [2.00;5.00] | 4.00 [2.00;6.00] | 4.00 [2.00;6.00] | 4.00 [2.00;5.00] | 0.177 |
| High *(≥6)* | 47 (18.0%) | 33 (29.5%) | 56 (25.9%) | 2 (6.25%) | **0.006** |
| Obesity (Heavy) | 62 (23.8%) | 25 (22.3%) | 75 (34.7%) | 6 (18.8%) | **0.016** |
| Hypertension | 160 (63.7%) | 78 (77.2%) | 144 (70.6%) | 17 (60.7%) | 0.061 |
| Atrial Fibrillation | 74 (28.4%) | 43 (38.4%) | 80 (37.0%) | 11 (34.4%) | 0.137 |
| Pulmonary Hypertension | 100 (61.3%) | 47 (63.5%) | 61 (48.4%) | 11 (50.0%) | 0.080 |
| Elderly (Age > 60) | 257 (98.5%) | 109 (97.3%) | 214 (99.1%) | 29 (90.6%) | **0.029** |
| Impaired Filling | 87 (85.3%) | 26 (83.9%) | 71 (83.5%) | 12 (92.3%) | 0.925 |
| **HFA-pEFF (numerical)** | 5.00 [4.00;6.00] | 5.00 [5.00;6.00] | 5.00 [4.00;5.00] | 4.00 [3.75;5.25] | **<0.001** |
| High *(≥5)* | 173 (66.3%) | 85 (75.9%) | 117 (54.2%) | 15 (46.9%) | **<0.001** |
| Functional |  |  |  |  | 0.527 |
| 1 | 63 (24.1%) | 31 (27.7%) | 65 (30.1%) | 8 (25.0%) |  |
| 2 | 198 (75.9%) | 81 (72.3%) | 151 (69.9%) | 24 (75.0%) |  |
| Morphological |  |  |  |  | **<0.001** |
| 0 | 57 (21.8%) | 18 (16.1%) | 52 (24.1%) | 11 (34.4%) |  |
| 1 | 76 (29.1%) | 25 (22.3%) | 96 (44.4%) | 10 (31.2%) |  |
| 2 | 128 (49.0%) | 69 (61.6%) | 68 (31.5%) | 11 (34.4%) |  |
| Biomarker |  |  |  |  | **<0.001** |
| 0 | 12 (4.60%) | 2 (1.79%) | 19 (8.80%) | 4 (12.5%) |  |
| 1 | 16 (6.13%) | 3 (2.68%) | 28 (13.0%) | 4 (12.5%) |  |
| 2 | 233 (89.3%) | 107 (95.5%) | 169 (78.2%) | 24 (75.0%) |  |
| Combined |  |  |  |  | **<0.001** |
| Both Low | 74 (28.4%) | 22 (19.6%) | 78 (36.1%) | 17 (53.1%) |  |
| High H_2_FpEF, Low HFA-pEFF | 14 (5.36%) | 5 (4.46%) | 21 (9.72%) | 0 (0.00%) |  |
| Low H_2_FpEF, High HFA-pEFF | 140 (53.6%) | 57 (50.9%) | 82 (38.0%) | 13 (40.6%) |  |
| Both High | 33 (12.6%) | 28 (25.0%) | 35 (16.2%) | 2 (6.25%) |  |
| Values are n(%). A P value of <0.05 was considered as statistically signiﬁcant.  HG-AS: high-gradient aortic stenosis; cLF-LG: classical low-flow, low-gradient; LF-LG pEF: low-flow, low-gradient with preserved ejection fraction; NF-LG: normal-flow, low-gradient. MI: myocardial infarction. | | | | | |

### **Supplementary Table 3:** Clusters and H_2_FPEF score and percentage of AF for different missing numbers thresholds.

| **Threshold** |  | **Cluster 1** | **Cluster 2** | **Cluster 3** | **Cluster 4** |
| --- | --- | --- | --- | --- | --- |
| 95% | N | 139 | 77 |  |  |
|  | H_2_FPEF | 3.40 | **5.30** |  |  |
|  | AF (%) | 17.27 | **72.73** |  |  |
| 90% | N | 77 | 139 |  |  |
|  | H_2_FPEF | **5.30** | 3.40 |  |  |
|  | AF (%) | **72.73** | 17.27 |  |  |
| 85% | N | 66 | 93 | 57 |  |
|  | H_2_FPEF | 3.65 | 3.51 | **5.49** |  |
|  | AF (%) | 22.73 | 21.51 | **78.95** |  |
| 80% | N | 92 | 66 | 58 |  |
|  | H_2_FPEF | 3.49 | 3.65 | **5.48** |  |
|  | AF (%) | 21.74 | 22.73 | **77.59** |  |
| 75% | N | 138 | 78 |  |  |
|  | H_2_FPEF | 3.41 | **5.24** |  |  |
|  | AF (%) | 17.39 | **71.79** |  |  |
| 70% | N | 67 | 22 | 75 | 52 |
|  | H_2_FPEF | 3.57 | **5.41** | 3.32 | 5.25 |
|  | AF (%) | 20.90 | 77.27 | 16.00 | **71.15** |
| 65% | N | 58 | 92 | 66 |  |
|  | H_2_FPEF | **5.48** | 3.49 | 3.65 |  |
|  | AF (%) | **77.59** | 21.74 | 22.73 |  |
| 60% | N | 64 | 88 | 64 |  |
|  | H_2_FPEF | **5.36** | 3.53 | 3.53 |  |
|  | AF (%) | **76.56** | 22.73 | 17.19 |  |
| 55% | N | 98 | 64 | 54 |  |
|  | H_2_FPEF | 3.55 | 3.58 | **5.61** |  |
|  | AF (%) | 24.49 | 18.75 | **81.48** |  |
| 50% | N | 99 | 64 | 53 |  |
|  | H_2_FPEF | 3.57 | 3.58 | **5.62** |  |
|  | AF (%) | 25.25 | 18.75 | **81.13** |  |
| 45% | N | 153 | 63 |  |  |
|  | H_2_FPEF | 3.52 | **5.41** |  |  |
|  | AF (%) | 21.57 | **74.60** |  |  |
| 40% | N | 54 | 62 | 100 |  |
|  | H_2_FPEF | **5.65** | 3.60 | 3.52 |  |
|  | AF (%) | **81.48** | 19.35 | 24.00 |  |
| 35% | N | 85 | 56 | 75 |  |
|  | H_2_FPEF | 3.55 | **5.55** | 3.56 |  |
|  | AF (%) | 23.53 | **78.57** | 21.33 |  |
| 30% | N | 59 | 157 |  |  |
|  | H_2_FPEF | **5.54** | 3.52 |  |  |
|  | AF (%) | **79.66** | 21.02 |  |  |
| 25% | N | 58 | 158 |  |  |
|  | H_2_FPEF | **5.52** | 3.54 |  |  |
|  | AF (%) | **79.31** | 21.52 |  |  |
| 20% | N | 116 | 100 |  |  |
|  | H_2_FPEF | **4.45** | 3.64 |  |  |
|  | AF (%) | **49.14** | 23.00 |  |  |
| 15% | N | 84 | 132 |  |  |
|  | H_2_FPEF | 3.90 | **4.18** |  |  |
|  | AF (%) | 30.95 | **40.91** |  |  |
| 10% | N | 114 | 102 |  |  |
|  | H_2_FPEF | 3.66 | **4.54** |  |  |
|  | AF (%) | 25.44 | **50.00** |  |  |
| 5% | N | 99 | 117 |  |  |
|  | H_2_FPEF | 4.02 | **4.12** |  |  |
|  | AF (%) | 32.32 | **41.03** |  |  |
| Threshold representing percentage of missing allowed. N: number of patients per cluster. H_2_FPEF: mean score per cluster. AF: percentage of atrial fibrillation in cluster. | | | | | |

**Supplementary Table 4:** Peri-procedural characteristics

|  | **Cluster 1** | **Cluster 2** |  |
| --- | --- | --- | --- |
|  | **N=77** | **N=139** | **p-value** |
| ***Peri-procedural characteristics*** |  |  |  |
| Transfemoral access | 57 (74.0%) | 120 (86.3%) | **0.039** |
| Expand |  |  | 0.077 |
| Balloon | 21 (29.6%) | 24 (17.8%) |  |
| Self | 50 (70.4%) | 111 (82.2%) |  |
| Femoral size | 15.0 (3.60) | 15.3 (3.43) | 0.703 |
| Implanted valve size | 27.6 (3.00) | 28.1 (2.93) | 0.262 |
| Multiple valves implanted | 1 (1.67%) | 4 (3.45%) | 0.662 |
| Use of embolic protection device | 48 (77.4%) | 93 (74.4%) | 0.786 |
| Pre-dilatation performed | 22 (36.1%) | 44 (36.4%) | 1.000 |
| Post-dilatation performed | 8 (12.9%) | 8 (6.96%) | 0.298 |
| Echo-guided puncture | 15 (93.8%) | 45 (88.2%) | 1.000 |
| Bicuspid aortic valve anatomy | 1 (1.85%) | 1 (0.91%) | 0.551 |
| Anaesthesia type |  |  | 0.060 |
| General Anaesthesia | 76 (98.7%) | 128 (92.1%) |  |
| Local Anaesthesia | 1 (1.30%) | 5 (3.60%) |  |
| Sedation | 0 (0.00%) | 6 (4.32%) |  |
|  |  |  |  |

**Supplementary Table 5:** Important echocardiographic parameters clusters (LASSO-Derived)

|  |  | Cluster 1  N=77 | | Cluster 2  N=139 | |  |
| --- | --- | --- | --- | --- | --- | --- |
|  | N | Mean (SD) | Mean Scaled SD | Mean (SD) | Mean Scaled SD | **p-value** |
| LAEDV (A4C) | 181 | 79.1 (26.6) | 0.929 | 35.4 (13.8) | -0.514 | **<0.001** |
| LA area (A4C) | 181 | 24.8 (5.42) | 0.927 | 14.6 (3.75) | -0.513 | **<0.001** |
| LA volume (Area-Length) (A4C) | 181 | 84.4 (27.9) | 0.914 | 38.2 (15.1) | -0.506 | **<0.001** |
| LA area (A2C) | 171 | 25.0 (6.37) | 0.899 | 13.5 (3.92) | -0.498 | **<0.001** |
| LAED volume index (A2C) | 101 | 46.1 (14.8) | 0.881 | 18.6 (7.88) | -0.488 | **<0.001** |
| LA width (A2C) | 179 | 47.2 (6.65) | 0.856 | 34.6 (5.36) | -0.474 | **<0.001** |
| LA volume (Area-Length) (A2C) | 171 | 88.4 (34.2) | 0.850 | 35.2 (15.9) | -0.471 | **<0.001** |
| LAES volume (biplane Area-Length) | 150 | 9.91 (2.58) | 0.839 | 6.18 (1.45) | -0.465 | **<0.001** |
| RA length (A4C) | 166 | 53.4 (7.81) | 0.787 | 39.6 (5.99) | -0.436 | **<0.001** |
| RA area (A4C) | 166 | 19.6 (5.77) | 0.783 | 10.9 (2.93) | -0.434 | **<0.001** |
| LA circumference (A2C) | 179 | 15.0 (2.14) | 0.780 | 10.9 (1.68) | -0.432 | **<0.001** |
| LA length (A2C) | 179 | 60.6 (8.85) | 0.766 | 44.9 (7.25) | -0.424 | **<0.001** |
| RA width (A4C) | 166 | 42.7 (7.29) | 0.741 | 32.3 (5.47) | -0.410 | **<0.001** |
| MV-E | 151 | 108 (22.7) | 0.640 | 80.7 (20.4) | -0.354 | **<0.001** |
| RA width (A4C) | 172 | 41.9 (6.53) | 0.617 | 33.8 (5.05) | -0.342 | **<0.001** |
| LV GLS (Basal infero-lateral) | 143 | -13.87 (7.14) | 0.590 | -21.63 (8.63) | -0.327 | **<0.001** |
| e’ mean | 98 | 7.98 (1.77) | 0.554 | 6.15 (1.69) | -0.307 | **<0.001** |
| LV GLS (Mid infero-septal) | 204 | -10.07 (5.19) | 0.504 | -13.84 (4.43) | -0.279 | **<0.001** |
| PASP | 126 | 43.4 (13.2) | 0.423 | 34.7 (13.2) | -0.234 | **<0.001** |
| LV area (A3C) | 138 | 21.0 (5.60) | -0.307 | 24.4 (5.75) | 0.170 | **0.001** |
| Mitral reg. (vena contracta) | 24 | 0.40 (0.17) | -0.363 | 0.38 (0.11) | 0.201 | 0.666 |
| LVED volume index (A3C) | 84 | 29.9 (10.3) | -0.393 | 35.3 (11.1) | 0.217 | **0.033** |
| Stroke volume LVOT (VTI) | 157 | 54.3 (16.2) | -0.463 | 71.2 (19.8) | 0.256 | **<0.001** |
| Mitral valve area | 151 | 2.98 (0.70) | -0.466 | 4.10 (1.18) | 0.258 | **<0.001** |
| Deceleration time (mitral inflow) | 100 | 177 (34.4) | -0.520 | 208 (47.1) | 0.288 | **<0.001** |
| LV circumference (A4C) | 205 | 16.5 (1.65) | -0.550 | 18.0 (1.56) | 0.305 | **<0.001** |
| LVOT VTI | 209 | 19.7 (4.97) | -0.590 | 25.1 (5.57) | 0.327 | **<0.001** |
| LA reservoir strain (A2C) | 157 | 9.68 (8.16) | -0.656 | 27.3 (11.7) | 0.364 | **<0.001** |
| LA reservoir strain (mean) | 136 | 9.40 (6.15) | -0.703 | 25.5 (7.96) | 0.390 | **<0.001** |
| LA reservoir strain (A4C) | 174 | 8.81 (5.18) | -0.736 | 22.7 (10.1) | 0.408 | **<0.001** |
| Table showing non-imputed data, sorted on importance.  A2C: Apical Two-Chamber View; A3C: Apical Three-Chamber View; A4C: Apical Four-Chamber View; GLS: Global Longitudinal Strain; LAED: Left Atrial End-Diastole; LAEDV: Left Atrial End-Diastolic Volume; LAESV: Left Atrial End-Systolic Volume; LV: Left Ventricle; LVED: Left Ventricular End-Diastole; LVOT: Left Ventricular Outflow Tract; MV-E: Mitral Valve Early Diastolic Filling Velocity; PASP: Pulmonary Artery Systolic Pressure; RA: Right Atrium; reg.: Regurgitation; VTI: Velocity Time Integral. | | | | | | |

**Supplementary Table 6:** Peri-procedural complications and outcomes

|  | **Cluster 1** | **Cluster 2** |  | |
| --- | --- | --- | --- | --- |
|  | **N=77** | **N=139** | **p-value** | |
| Complications during procedure |  |  |  | |
| Conversion to open surgery | 0 (0.00%) | 0 (0.00%) | . | |
| Percutaneous closure device failure | 4 (7.84%) | 5 (4.81%) | 0.478 | |
| Placement of peripheral stent | 2 (4.55%) | 5 (5.26%) | 1.000 | |
| New cardiac surgery during admission | 2 (2.60%) | 1 (0.74%) | 0.297 | |
| Valve embolization | 0 (0.00%) | 0 (0.00%) | . | |
| Stroke during procedure | 2 (2.60%) | 10 (7.19%) | 0.220 | |
| Stroke classification |  |  | 1.000 | |
| Haemorrhagic | 0 (0.00%) | 2 (20.0%) |  | |
| Ischaemic | 2 (100%) | 8 (80.0%) |  | |
| Vascular complication | 14 (18.2%) | 21 (15.1%) | 0.693 | |
| Severity of vascular complication |  |  | 0.474 | |
| Major Vascular Complications | 5 (35.7%) | 5 (23.8%) |  | |
| Minor Vascular Complications | 9 (64.3%) | 16 (76.2%) |  | |
| Aortic dissection | 1 (1.32%) | 3 (2.22%) | 1.000 | |
| Cardiac tamponade | 1 (1.32%) | 5 (3.70%) | 0.422 | |
| Annulus rupture | 0 (0.00%) | 0 (0.00%) | . | |
| Coronary obstruction | 0 (0.00%) | 0 (0.00%) | . | |
| New Pacemaker Implantation | 13 (16.9%) | 14 (10.1%) | 0.217 | |
| Acute kidney injury (AKIN classification) |  |  | 0.340 | |
| No kidney failure | 65 (85.5%) | 124 (89.9%) |  | |
| Stage 1 | 4 (5.26%) | 9 (6.52%) |  | |
| Stage 2 | 5 (6.58%) | 4 (2.90%) |  | |
| Stage 3 | 2 (2.63%) | 1 (0.72%) |  | |
| Bleeding (peri-procedural) | 19 (24.7%) | 24 (17.3%) | 0.259 | |
| Bleeding severity |  |  | 0.208 | |
| Life Threatening or Disabling Bleeding | 3 (15.8%) | 5 (20.8%) |  | |
| Major Bleeding | 8 (42.1%) | 4 (16.7%) |  | |
| Minor Bleeding | 8 (42.1%) | 15 (62.5%) |  | |
| Location bleeding |  |  | 0.286 | |
| Access site | 2 (40.0%) | 4 (80.0%) |  | |
| Gastrointestinal | 1 (20.0%) | 0 (0.00%) |  | |
| Other | 2 (40.0%) | 0 (0.00%) |  | |
| Retroperitoneal | 0 (0.00%) | 1 (20.0%) |  | |
| Aortic regurgitation/PVL grade *(during procedure, on angiogram)* | |  | 0.332 | |
| 0 | 35 (45.5%) | 73 (52.5%) |  | |
| 1 | 33 (42.9%) | 45 (32.4%) |  | |
| 2 | 3 (3.90%) | 9 (6.47%) |  | |
| 3 | 1 (1.30%) | 0 (0.00%) |  | |
| Not tracked | 5 (6.49%) | 12 (8.63%) |  | |
| **Outcomes** |  |  |  | |
| Aortic regurgitation grade *(on TTE, <7d after TAVI)* |  |  | 0.596 | |
| 0 | 50 (71.4%) | 92 (70.2%) |  | |
| 1 | 17 (24.3%) | 29 (22.1%) |  | |
| 2 | 3 (4.29%) | 4 (3.05%) |  | |
| 3 | 0 (0.00%) | 2 (1.53%) |  | |
| Not possible to grade | 0 (0.00%) | 4 (3.05%) |  | |
|  |  |  |  | |
|  | | | |  |

### **Supplementary Table 7:** Outcomes complete cohort

|  | **HG-AS** | **cLF-LG** | **LF-LG pEF** | **NF-LG** | **p** |
| --- | --- | --- | --- | --- | --- |
|  | **N=261** | **N=112** | **N=216** | **N=32** |  |
| **1-year** |  |  |  |  |  |
| Cardiovascular mortality | 16 (6.13%) | 11 (9.82%) | 19 (8.80%) | 5 (15.6%) | 0.198 |
| All-cause mortality | 29 (11.1%) | 22 (19.6%) | 28 (13.0%) | 7 (21.9%) | 0.079 |
| HF-Hospitalisation | 31 (11.9%) | 20 (17.9%) | 24 (11.1%) | 3 (9.38%) | 0.328 |
| Stroke | 13 (4.98%) | 5 (4.46%) | 20 (9.26%) | 1 (3.12%) | 0.207 |
| MI | 3 (1.15%) | 4 (3.57%) | 3 (1.39%) | 0 (0.00%) | 0.388 |
| Lack of symptom improvement | 38 (15.0%) | 24 (22.2%) | 55 (25.5%) | 9 (30.0%) | **0.020** |
| Combined endpoint**^†^** | 25 (11.6%) | 16 (18.4%) | 29 (17.5%) | 7 (26.9%) | 0.093 |
| **5-year** |  |  |  |  |  |
| Cardiovascular mortality | 65 (24.9%) | 43 (38.4%) | 64 (29.6%) | 13 (40.6%) | **0.033** |
| All-cause mortality | 93 (35.6%) | 55 (49.1%) | 103 (47.7%) | 19 (59.4%) | **0.005** |
| HF- Hospitalisation | 59 (22.6%) | 41 (36.6%) | 52 (24.1%) | 7 (21.9%) | **0.031** |
| Stroke | 23 (8.81%) | 9 (8.04%) | 30 (13.9%) | 5 (15.6%) | 0.175 |
| MI | 7 (2.68%) | 10 (8.93%) | 6 (2.78%) | 1 (3.12%) | **0.042** |
| Values are n(%). A P value of <0.05 was considered as statistically signiﬁcant. Combined endpoint: both lack of symptom improvement and an adverse event.  CV: cardiovascular; HF: heart failure; HG-AS: high-gradient aortic stenosis; cLF-LG: classical low-flow, low-gradient; LF-LG pEF: low-flow, low-gradient with preserved ejection fraction; NF-LG: normal-flow, low-gradient. MI: myocardial infarction. | | | | | |

**Supplementary Table 8:** Flow-types validation and derivation cohort

|  | **2009-2020** | **2020-2024** | P-value |
| --- | --- | --- | --- |
|  | **N=628** | **N=375** |  |
| Flow-Type |  |  | **0.003** |
| HG-AS | 261 (41.6%) | 196 (52.3%) |  |
| cLF-LG | 112 (17.8%) | 41 (10.9%) |  |
| LF-LG pEF | 216 (34.4%) | 111 (29.6%) |  |
| NF-LG | 32 (5.10%) | 20 (5.33%) |  |
| Discongruent | 7 (1.11%) | 7 (1.87%) |  |

**Supplementary Table 9:** Baseline characteristics derivation and internal validation cohort

|  | **2009-2020**  **(Derivation)** | **2020-2024**  **(Internal validation)** | **p-value** |
| --- | --- | --- | --- |
|  | **N=216** | **N=111** |  |
| Age | 78.8 (7.19) | 81.0 (6.15) | **0.005** |
| NYHA 3/4 | 189 (87.5%) | 75 (67.6%) | **<0.001** |
| COPD | 67 (31.0%) | 20 (18.0%) | **0.017** |
| CAD | 129 (59.7%) | 44 (40.0%) | **0.001** |
| PCI | 91 (42.1%) | 18 (16.2%) | **<0.001** |
| Na | 139 (3.80) | 138 (3.33) | **0.035** |
| AVMean | 28.9 (7.46) | 31.4 (7.22) | **0.004** |
| MR Vena Contracta | 0.39 (0.14) | 0.29 (0.06) | **0.004** |
| SVI | 24.2 (5.55) | 25.7 (5.50) | **0.016** |
| LVGls | -16.38 (3.96) | -17.45 (3.81) | **0.018** |
| Betablokker | 147 (68.1%) | 61 (55.5%) | **0.034** |
| MRA | 21 (9.72%) | 20 (19.2%) | **0.027** |
| VKA | 63 (29.2%) | 20 (18.2%) | **0.044** |
| NOAC | 20 (9.30%) | 22 (20.0%) | **0.011** |
| H_2_FpEF | 4.07 (2.15) | 3.38 (1.91) | **0.003** |
| HFA-pEFF | 4.47 (1.17) | 4.74 (1.10) | **0.040** |
| H_2_FpEF x HFA-pEFF: |  |  | **0.002** |
| Both Low | 78 (36.1%) | 54 (48.6%) |  |
| High H2FpEF, Low HFApEFF | 21 (9.72%) | 16 (14.4%) |  |

**Supplementary Table 10:** Clusters internal validation cohort

|  | **Cluster 1 (VAL)** | **Cluster 2 (VAL)** |  |
| --- | --- | --- | --- |
|  | **N=55** | **N=56** | **p-value** |
| **Demographics** |  |  |  |
| Age | 81.3 (5.80) | 80.6 (6.51) | 0.570 |
| Sex (Female) | 24 (43.6%) | 40 (71.4%) | **0.006** |
| AF | 31 (60.8%) | 5 (9.62%) | **<0.001** |
| NTpro-BNP (pre) | 2286 (2872) | 1240 (2520) | **0.045** |
| **HFpEF Scores** |  |  |  |
| H_2_FpEF (numerical) | 5.00 [3.00;7.00] | 3.00 [2.00;4.00] | **<0.001** |
| High *(≥6)* | 23 (41.8%) | 3 (5.36%) | **<0.001** |
| HFA-pEFF (numerical) | 5.00 [4.00;6.00] | 5.00 [4.00;5.00] | **0.011** |
| High *(≥5)* | 39 (70.9%) | 31 (55.4%) | 0.133 |
| Combined |  |  | **<0.001** |
| Both Low | 21 (38.2%) | 29 (51.8%) |  |
| High H_2_FpEF, Low HFA-pEFF | 18 (32.7%) | 2 (3.57%) |  |
| Low H_2_FpEF, High HFA-pEFF | 11 (20.0%) | 24 (42.9%) |  |
| Both High | 5 (9.09%) | 1 (1.79%) |  |
| VAL: validation. | | | |

**Supplementary Table 11:** Baseline and clusters external validation cohort

|  | **Total cohort** | **n** | **Clusters** | |  |
| --- | --- | --- | --- | --- | --- |
|  |  | **N** | **1** | **2** |  |
|  | N=173 |  | **N=93** | **N=80** | **P-value** |
| Age (y) | 82.4 (5.82) | 173 | 82.2 (6.05) | 82.5 (5.58) | 0.755 |
| Sex (female) | 96 (55.5%) | 173 | 50 (53.8%) | 46 (57.5%) | 0.734 |
| BMI (kg/m²) | 26.7 (5.09) | 173 | 27.1 (6.03) | 26.2 (3.67) | 0.223 |
| EuroScore-2 (log) | 4.56 (3.97) | 170 | 4.84 (3.30) | 4.23 (4.63) | 0.336 |
| H2FPEF score | 5.42 (1.81) | 167 | 6.26 (1.53) | 4.46 (1.63) | **<0.001** |
| HFA-PEFF score | 5.32 (1.02) | 100 | 5.81 (0.48) | 4.67 (1.19) | **<0.001** |
| History AF | 105 (62.9%) | 167 | 75 (84.3%) | 30 (38.5%) | **<0.001** |
| History of hypertension | 151 (87.8%) | 172 | 78 (84.8%) | 73 (91.2%) | 0.290 |
| AVA (cm²) | 0.71 (0.15) | 173 | 0.71 (0.15) | 0.71 (0.15) | 0.953 |
| Mean AV gradient (mmHg) | 28.0 (7.41) | 173 | 27.1 (7.27) | 29.0 (7.49) | 0.096 |
| LVEF (%) | 57.1 (4.27) | 173 | 56.7 (4.50) | 57.6 (3.95) | 0.143 |
| LV mass index (g/m²) | 112 (29.9) | 108 | 106 (32.2) | 93.4 (19.8) | **0.002** |
| LA volume index (mL/m²) | 47.2 (26.9) | 103 | 49.8 (26.6) | 32.7 (6.38) | **<0.001** |
| PASP (mmHg) | 48.6 (16.5) | 52 | 41.8 (14.3) | 33.7 (2.67) | **<0.001** |
| e’ mean | 7.03 (1.98) | 128 | 7.63 (1.73) | 6.38 (1.40) | **<0.001** |
| MV-E | 106 (38.6) | 133 | 118 (35.2) | 84.9 (23.6) | **<0.001** |
| SVI (mL/m²) | 28.8 (5.05) | 173 | 27.5 (5.42) | 30.2 (4.19) | **<0.001** |
| LA reservoir str (%) | 14.2 (7.86) | 139 | 11.8 (6.10) | 19.5 (6.67) | **<0.001** |
| LV global strain (%) | -14.28 (4.15) | 147 | -13.28 (3.69) | -16.12 (3.58) | **<0.001** |
| NT-proBNP (ng/L) | 1510 (2434) | 165 | 1971 (2495) | 983 (2264) | **0.008** |
| Agatston score | 2089 (1226) | 136 | 2028 (1007) | 2164 (1456) | 0.538 |
| High Score  *(>1200 (F) or >2000 (M))* | 88 (64.7%) | 136 | 53 (70.7%) | 35 (57.4%) | 0.152 |
| Creatinine (mg/dl) | 1.14 (0.37) | 173 | 1.17 (0.36) | 1.10 (0.38) | 0.180 |
| Loop diuretic use | 108 (62.8%) | 172 | 66 (71.7%) | 42 (52.5%) | **0.014** |
| Aspirin use | 58 (33.5%) | 173 | 24 (25.8%) | 34 (42.5%) | **0.031** |
|  | | | | | |

### **Supplementary Figure 1: Me**thods external validation

*1) Cluster on >300 echo variables from UMCG cohort, 2) Resulting in 2 clusters, 3) From this cohort, retract most important parameters (30), 4) Select similar variables in Munich Cohort, 5) Determine the ‘centroids’ in UMCG cohort, 6) Impute the Munich variables with the median of UMCG cohort, 7) Scale and standardize the Munich cohort, similar to the UMCG cohort, 8) Assign the Munich cohort with the closest Euclidean distance from original centroids 9) This results in again 2 clusters*

### **Supplementary Figure 2:** Flow-diagram Inclusion, regular flow-types

Flow diagram showing included patients, stratified by regular flow-types.

† Showing percentage of group from the previous step.

### **Supplementary Figure 3:** Course of NT-proBNP during 1-year follow-up

Plots showing NT-proBNP (left) and log-NT-proBNP (right) levels pre-procedural (pre), 30 days (30d), 6 months (6m) and 12 months (12m) after TAVR (transcatheter aortic valve replacement).
cLF-LG: classical low-flow, low gradient; HG-AS: high-gradient aortic stenosis, NF: normal-flow, low gradient aortic stenosis
